# Nanoelectromechanical relay without pull-in instability for high-temperature non-volatile memory

**DOI:** 10.1038/s41467-020-14872-2

**Published:** 2020-03-04

**Authors:** Sunil Rana, João Mouro, Simon J. Bleiker, Jamie D. Reynolds, Harold M. H. Chong, Frank Niklaus, Dinesh Pamunuwa

**Affiliations:** 1 Department of Electrical and Electronic Engineering, University of Bristol, Bristol, BS8 1UB UK; 2 Division of Micro and Nanosystems, KTH Royal Institute of Technology, Stockholm, 114 28 Sweden; 3 School of Electronics and Computer Science, University of Southampton, Southampton, SO17 1BJ UK

**Keywords:** Electrical and electronic engineering, Electronic devices, NEMS

## Abstract

Emerging applications such as the Internet-of-Things and more-electric aircraft require electronics with integrated data storage that can operate in extreme temperatures with high energy efficiency. As transistor leakage current increases with temperature, nanoelectromechanical relays have emerged as a promising alternative. However, a reliable and scalable non-volatile relay that retains its state when powered off has not been demonstrated. Part of the challenge is electromechanical pull-in instability, causing the beam to snap in after traversing a section of the airgap. Here we demonstrate an electrostatically actuated nanoelectromechanical relay that eliminates electromechanical pull-in instability without restricting the dynamic range of motion. It has several advantages over conventional electrostatic relays, including low actuation voltages without extreme reduction in critical dimensions and near constant actuation airgap while the device moves, for improved electrostatic control. With this nanoelectromechanical relay we demonstrate the first high-temperature non-volatile relay operation, with over 40 non-volatile cycles at 200 ^∘^C.

## Abstract (web summary)

Designing reliable, scalable and energy efficient data storage systems that can operate in extreme temperatures, remains a challenge. Here, the authors demonstrate a nanoelectromechanical relay that does not exhibit pull-in instability for reliable reprogrammable non-volatile memory operation.

## Introduction

Nanoelectromechanical (NEM) relays have zero-off state current and a steep subthreshold slope^1–6^ and can operate at very high temperatures^7^. Thus, they have potential to replace transistors in applications that require very high-energy efficiency and/or harsh-environment capability. To realise general-purpose NEM relay-based computing, logic and memory circuits are required^8^. Logic is generally implemented using relays that pull out of contact to the ‘off’ state upon removal of the gate voltage. Non-volatile memory requires relays that retain the switched state when power is switched off. Electrostatically operated NEM relays reported in the literature for both volatile operation^5,9,6,7,10–12^ and non-volatile operation^12–14^ have architectures including a beam anchored at one end (cantilever), both ends (bridge), the centre (see-saw) and at four corners (crab leg), with different types of hinges. When actuated, the beam moves under an electrostatic force to make contact with a stationary electrode to establish the ‘on’ state. As the beam moves, the airgap between the actuation electrode (gate) and beam (source) rapidly reduces while the capacitance increases. At a critical voltage called the pull-in voltage, the electrostatic force becomes much greater than the opposing spring force and the beam snaps in. We refer to this electromechanical instability caused by the applied electrostatic force as pull-in instability. In the case of a parallel-plate capacitor with one plate on a spring that moves orthogonally towards the other plate, pull-in occurs when the plate moves a distance equal to 1/3rd of the rest-state gap. Pull-in instability can be avoided by increasing the contact dimple size to ensure the beam lands on it before the point of instability is reached. This, however, requires the beam-to-gate airgap to be up to 3×  the contact separation depending on the architecture, which increases the actuation voltage. Pull-in instability limits reliability and also causes significant fabrication challenges, as extremely small airgaps, of the range of a few nm to tens of nm^9,12^, are required to achieve low voltages. Further, contacts deteriorate faster with heavy impacts, of particular concern in non-volatile relays that use contact adhesion forces to achieve bistability. Thus, only a very small number of reprogramming cycles have been demonstrated for bistable relays to date^13–16^, which highlights the challenge in realising reliable non-volatile operation.

Here we report the first electrostatically actuated NEM relay that eliminates pull-in instability without restricting the dynamic range of motion. In contrast to conventional architectures, our relay has a semicircular beam that rotates clockwise or anticlockwise around a soft hinge due to simultaneous actuation of a pair of gates in an arrangement of four gates. A near constant actuation airgap is maintained throughout relay operation, eliminating the electromechanical stability issues in conventional electrostatic actuation schemes. Using this relay we demonstrate the first high-temperature non-volatile operation of a NEM relay, with 42 cycles at 200 °C. We also demonstrate data retention over more than 6 months, the highest number of reprogramming cycles to date under any environmental conditions, actuation voltages as low as 1.6 V with a 120 nm actuation airgap, and provide a comprehensive model for the moment-driven movement and non-volatile operation of the relay. These results demonstrate the potential of the reported NEM relays to realise high-temperature capable, reprogrammable non-volatile memory.

## Results

### Demonstration of non-volatile operation

**Fig. 1:**
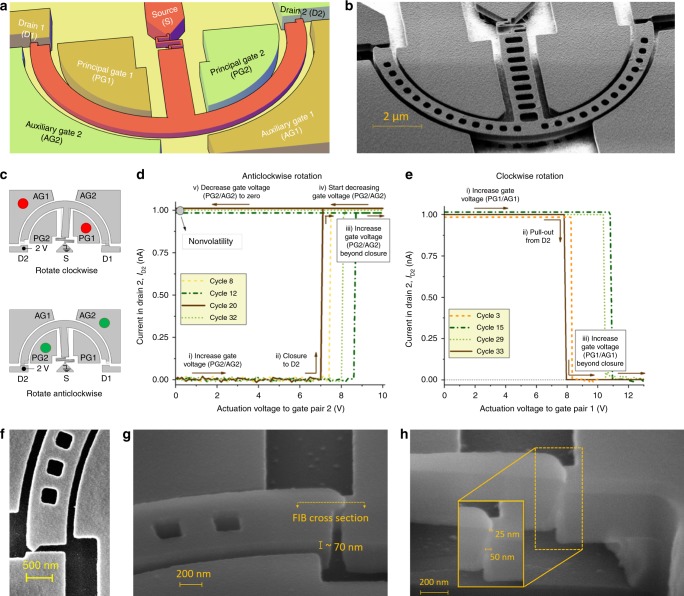
Moment-driven relay with in-plane, quad-gate architecture. **a** Sketch and **b** micrograph of bistable nanorelay with a serpentine hinge, 120 nm gate-to-beam airgap, 100 nm rest-state contact gap and 80 nm hinge width. **c** Actuation patterns to switch relay with positive hinge offset (i.e. hinge anchor point above baseline diameter of beam semicircle) in the experiments conducted at high-temperature in a vacuum ambient. The coloured circles indicate connection of source measurement units; top (red) is used to rotate clockwise, while bottom (green) is used to rotate anticlockwise. **d**, **e** Examples of anticlockwise and clockwise rotation cycles at 200 °C of a nano relay with positive hinge offset 0.4 μm, where a dc bias is applied to D2, causing a current *I*_D2_ to flow through it and the beam (source, S) on switch closure. Thus, for anticlockwise rotations, closure is indicated by the sudden increase in *I*_D2_; for clockwise rotations pull-out from D2 is monitored, indicated by the sudden decrease in *I*_D2_. The beam electrode remains in contact with one of the drains after actuation voltages are decreased to zero. In clockwise rotations the actuation electrostatic force needs to overcome adhesion forces, as well as electrostatic forces resulting from the drain-source bias, resulting in a higher pull-out voltage. **f** Close-up of beam electrode in contact with D2, showing near constant inner and outer actuation airgaps. **g** Close up of tip in contact with D1 showing a layer of Ti on top. **h** Sectioned view of **g**. The total sidewall thickness across both contacting surfaces vary from  ~25 nm at top to  ~50 nm at middle.

Our proposed NEM relay consists of a semicircular beam that rotates in plane, and is actuated by applying a potential between the beam and a pair of gates (see Fig. 1a, b). The actuation pattern to rotate the relay in a given direction depends on whether the hinge anchor point is above (positive offset) or below (negative offset) the baseline diameter of the beam semicircle; the following description is for a relay with a positive hinge offset. Driving principal (i.e. inner) gate 1 and auxiliary (i.e. outer) gate 1 to the same voltage, with the beam grounded, results in a net clockwise rotational moment that causes the beam to rotate and land on drain 1 (Fig. 1c, top); driving principal gate 2 and auxiliary gate 2 causes the beam to rotate in the opposite direction and land on drain 2 (Fig. 1c, bottom). Simultaneous actuation via two diagonally opposite gates is key to reducing translational movement and maintaining a near constant actuation airgap as the beam rotates. This arrangement almost completely eliminates the changes in capacitance that cause pull-in instability in previously reported NEM relays. When the device is in contact with the drain, a current can flow between the source and drain. When operated as a bistable device the beam remains in the switched state through adhesion forces between the contacting surfaces on the drain and beam tip, when the actuation voltage is removed. Thus, the NEM relay serves as a non-volatile memory cell that can be programmed to one of two states (‘1’ or ‘0’) to realise larger non-volatile memories that retain state when power is switched off.

For clarity, the term closure is used to refer to the beam tip making contact with a drain in our relay. The term pull-in is only used in reference to conventional relay architectures, to describe the beam snapping in. Closure can occur starting with the beam in the neutral state not connected to either drain, or in contact with one of the drain electrodes. The term programming voltage is used to refer to the voltage required to actuate the relay from a neutral position. Reprogramming voltage refers to the voltage required to switch the relay to land on one of the drains when the beam starts off being in contact with the opposite drain. Finally, the term pull-out is used to refer to the beam overcoming the adhesion forces at the tip and pulling away from the contact, upon application of the pull-out voltage.

In order to demonstrate the moment-driven operation of the relay and reprogrammable non-volatile behaviour, we fabricated several nanoscale NEM relay prototypes with serpentine (Fig. 1b) and straight (Supplementary Fig. 2) hinges having hinge offsets of 0.4, 1.2 and  −1.0 μm and different contact sizes (see “Methods” section). These relays were cycled for 2 up to 20 cycles at room temperature under atmospheric conditions and subsequently stored for ~6 months under the same environmental conditions to evaluate data retention. The NEM relays successfully retained the last stored state over the duration in storage and a selection (covering the different hinge types and offsets described above) were reprogrammed in subsequent experiments at 200 °C in vacuum.

Cycling consists of repetitively rotating the device anticlockwise and clockwise until the device fails. The initial clockwise cycle is considered the first cycle. Anticlockwise reprogramming cycles start with the beam tip in contact with drain 1 (D1), and are measured by applying the actuation pattern shown in Fig. 1c, bottom, with a dc bias applied to drain 2 (D2). A sample of four of these cycles are shown in Fig. 1d for a relay with a serpentine hinge having an offset of 0.4 μm, where the waveforms demonstrate the following regimes: (i) The actuation voltage to gate pair 2 (PG2/AG2) is increased; (ii) when the actuation voltage on gate pair 2 reaches the closure voltage, the beam tip makes contact with drain 2 (D2), shown by the step increase in the current *I*_D2_ flowing through D2 and the beam (source, S); (iii) the actuation voltage on gate pair 2 is increased by around 2 V beyond the closure voltage; (iv) the actuation voltage on gate pair 2 is then decreased, with the drain current continuing to flow all the while; (v) the actuation voltage on gate pair 2 reaches zero, and the current through D2 continues to flow at the set current compliance, demonstrating non-volatility. These regimes are illustrated by the arrows in Fig. 1d for the waveform corresponding to cycle 20. The other cycles shown in the plot have exactly the same behaviour.

After every anticlockwise rotation, the beam tip is in contact with D2. Next, the relay is rotated clockwise by applying the actuation pattern shown in Fig. 1c, top, with the bias being maintained on the same drain, D2. A sample of four of these cycles are shown in Fig. 1e. Here, the following regimes can be identified in the waveforms: (i) the actuation voltage to gate pair 1 (PG1/AG1) is increased; (ii) when the actuation voltage on gate pair 1 reaches the pull-out voltage, the beam tip breaks contact with drain 2 (D2), shown by the step decrease in the current *I*_D2_, immediately followed by closure to the opposite drain D1 (This relay operates in the regime where the pull-out voltage is greater than the programming voltage, as explained in detail in section “Mechanical stability and regions of bistable operation”.) (contact is verified by a subsequent *I*–*V* curve); (iii) the actuation voltage on gate pair 1 is increased by around 2 V beyond the pull-out voltage.

Following a clockwise cycle, the gate probes are switched from gate pair 1 to to gate pair 2, to resume the next anticlockwise cycle. The anticlockwise and clockwise rotations are given unique sequential identifiers, so the first clockwise rotation is termed cycle 1, the next anticlockwise rotation cycle 2, the subsequent clockwise rotation cycle 3 and so on, as an anticlockwise rotation has to be followed by a clockwise rotation and vice versa. The on-current started to deteriorate in cycles 35 and 36, and opening and closing events could be monitored for a total of 42 cycles; afterwards, the switching events could not be detected electrically without increasing the drain bias. The relay continued to cycle mechanically until the experiments were halted.

We investigated the contribution of the drain bias to the adhesion force by consistently biasing the same drain (drain 2) for clockwise and anticlockwise actuation patterns. Thus, the actuating electrostatic force for clockwise rotations (actuation pattern at the top of Fig. 1c, red pattern) has to overcome surface adhesion forces, as well as any electrostatic forces resulting from the drain bias. It can be seen that the pull-out voltage at drain 2 (Fig. 1e) was consistently  ~1–2 V higher than the pull-out voltage at drain 1 (Fig. 1d). It seems quite likely that the drain and source make contact through a few surface asperities, with nm or angstrom scale airgaps surrounding the asperities. Thus, a high bias voltage likely results in a strong electrostatic field across these very small airgaps between the contacts and a non-negligible electrostatic force between the drain and source surfaces, notwithstanding the small area.

The semicircular beam and rotational mode of operation in the proposed relay results in a near constant actuation airgap in any state. A NEM relay after anticlockwise rotation (i.e. closure to drain 2) is shown in Fig. 1f. As can be seen, the inner and outer airgaps remain nearly constant. A close-up of the beam electrode in contact with drain 1 is shown in Fig. 1g. The relays have a layer of thermally evaporated Ti that is 70–80 nm thick on top. A cross-sectional view of the tip in contact with the drain is shown in Fig. 1h, indicating a total sidewall coverage comprising the layers on both the tip and the drain of between 25 and 50 nm. The contact resistance in these experiments was measured in *I*–*V* sweeps to be around 600 MΩ before contact degradation occurred. A thicker layer of metal and more conformal sidewall deposition should result in a lower contact resistance. We discuss alternative contact materials in section “Discussion”.

Finally, in order to gain further understanding of the role of hinge stiffness on programming and reprogramming voltages, we have fabricated and characterised a range of designs with a straight hinge. A straight hinge is stiffer than a serpentine hinge and the measurements of devices with both types of hinges provided more data to validate our models for moment-driven actuation and bistable operation. The results of these experiments, conducted in an open atmosphere at room temperature, are described in Supplementary Note 3 and shown in Supplementary Fig. 2.

### Demonstration of moment-driven operation

**Fig. 2:**
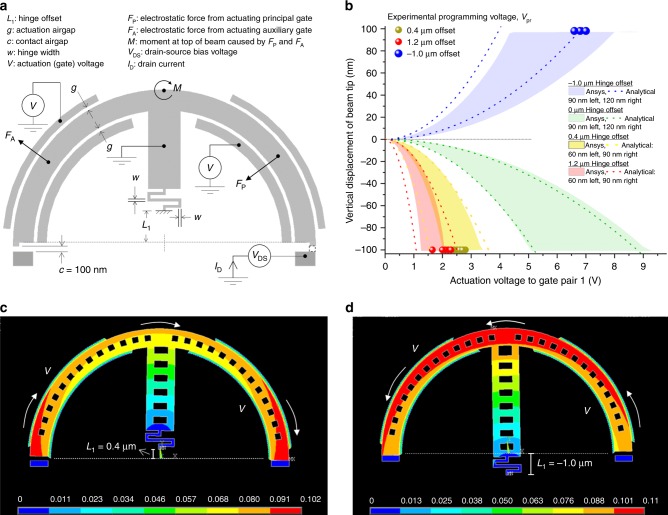
Forces and rotation moments in relay with serpentine hinge. **a** Relay with a positive hinge offset *L*_1_, defined as the vertical displacement of the hinge anchor point from the geometric centre of the circular beam. A negative hinge offset would result in the hinge anchor point being situated below the centre. When a voltage *V* is applied simultaneously on the principal and auxiliary gate pair 1, the resultant electrostatic fields across the two respective airgaps produce radially directed forces as shown. These forces resolve to realise a net clockwise rotational moment for positive hinge offsets, and an anticlockwise rotational moment for negative hinge offsets. **b** Measured programming voltages (circles) for different hinge offsets where the beam tip moves down 100 nm vertically to make contact with the drain. Multiple devices with the same hinge offset were tested with the differences in programming voltage due to fabrication non-uniformities across the die. Also shown are the predictions from the analytical model (dashed lines, model described in Supplementary Note 1 and Supplementary Fig. 1) and Ansys finite-element model (bands) for a range of hinge widths. Other than the hinge offsets, the geometry of the devices are identical and shown in Supplementary Table 1. **c**, **d** Ansys simulation of the total displacement for different hinge offsets of otherwise identical devices where the device is actuated in both instances by driving principal and auxiliary gate pair 1. The colour bar represents vertical displacement with red corresponding to 100 nm, the drain-to-beam-tip airgap.

Insight into the unique moment-driven behaviour and rotational motion of the beam in our NEM relay has been obtained by constructing analytical and finite-element models. The full analytical model is described in Supplementary Note 1 and the principle of operation is that a voltage applied to a principal and auxiliary gate pair results in electrostatic forces distributed radially across the respective airgaps (see Fig. 2a for the forces resulting from actuating gate pair 1). These forces combine to create a rotational moment that is a function of the position of the hinge anchor point with respect to the geometric centre of the beam, which we define as the hinge offset (*L*_1_ in Fig. 2a). For a positive offset, i.e. the anchor point is above the geometric centre, the net moment for actuation via gate pair 1 is clockwise; actuation via gate pair 2 results in an anticlockwise moment. For a negative offset, the directions of rotation of the moments are reversed; i.e. actuation via gate pair 1 results in an anticlockwise moment while actuation via gate pair 2 results in a clockwise moment. This is verified by finite-element simulations shown in Fig. 2c, d.

To investigate the influence of the hinge offset on the programming voltage, relays with three different offsets were fabricated. Measurements of the programming voltage for these NEM relays resulting from actuation via gate pair 1 are shown in Fig. 2b, along with finite-element and analytical model predictions. Increasing the hinge offset results in a greater moment and lower programming voltage, which can be seen, for example, by comparing the voltages for the nanorelays with 0.4 and 1.2 μm hinge offsets to achieve a negative vertical displacement of 100 nm (i.e. the rest-state contact separation) for clockwise rotation. Increasing the hinge offset however increases the eccentricity of motion which in turn causes the trajectory of the beam to deviate from a true circle. A negative offset results in anticlockwise rotation for the same actuation pattern, shown as a positive displacement of 100 nm in Fig. 2b. The different programming voltages for devices with identical hinge offsets seen in Fig. 2b appear to be a consequence of different hinge widths resulting from fabrication non-uniformities across the die. The hinge width for all of these characterised nanorelays was nominally designed to be 120 nm. However, we observed variation depending on their position in the die. In contrast, the actuation airgap was relatively unchanged from the nominal design dimension of 120 nm. This was due to overetch in more exposed areas, such as the hinge. The uncertainty in the final hinge width explains the dispersion in measured programming voltages for similar devices.

The simulated tip displacements as a function of actuation voltage are plotted in Fig. 2b, with the bands representing the finite-element model and the dotted lines the analytical model. Good agreement between the analytical and finite-element models and between the measurements and the models (please see Supplementary Note 1 for details of the analytical model) can be seen. It is noteworthy that we recorded programming voltages as low as 1.6 V without any particular focus on minimising the programming voltage. Finally a transient finite-element simulation was carried out to estimate the mechanical switching time of the NEM relays by applying a step input slightly higher than the programming voltage to gate pair 1 and monitoring the movement of the tip. This indicated the mechanical latency of the NEM relays with 1.2 μm hinge offset to be 511 and 954 ns for straight and serpentine hinge designs, respectively.

### Mechanical stability and regions of bistable operation

The non-volatile behaviour of our NEM relay has been modelled as follows. When the device experiences closure and the source and drain contacts touch, adhesion forces between the two contacting surfaces arise. Upon deactuation, if the surface adhesion force, *F*_adh_, is less than the elastic spring force in the hinge, *F*_spr_, the device pulls out and exhibits volatile behaviour. If, on the other hand, *F*_adh_ > *F*_spr_, the relay stays closed and exhibits non-volatile behaviour. In order to force apart the electrodes in contact and reprogramme non-volatile devices, an actuation voltage is applied to the opposite set of gates to create a rotation moment in the opposite direction. The pull-out event can be modelled by considering the balance of moments at the point of pull-out (e.g. refs. ^17,18^) and can occur in two distinct ways. The voltage required to break the contact, *V*_po_, i.e. pull-out voltage, can be lower or higher than the voltage required to rotate the circular beam from the neutral position towards the drain electrode, *V*_pr_, i.e. programming voltage. If *V*_po_ < *V*_pr_, the device pulls out but the actuation moment is insufficient to cause it to rotate fully to the opposite drain. As the actuation voltage is progressively increased and reaches *V*_pr_, the beam tip lands on the opposite drain. If, on the other hand, *V*_po_ > *V*_pr_, pull-out occurs, immediately followed by closure to the opposite drain. Both types of reprogramming behaviour were monitored and observed in experiments (see Supplementary Note 3 and Supplementary Fig. 2 for latter and Supplementary Note 4 and Supplementary Fig. 3 for former). In the regime where *V*_po_ > *V*_pr_, the over-drive increases the impact force on the source and drain contacts at closure, which can result in a firmer contact and larger effective area. This in turn can result in a higher adhesion force, requiring a higher *V*_po_ that then repeats and hastens the mechanical wear of the contacts (see for example, Supplementary Note 3 and Supplementary Fig. 2).

**Fig. 3:**
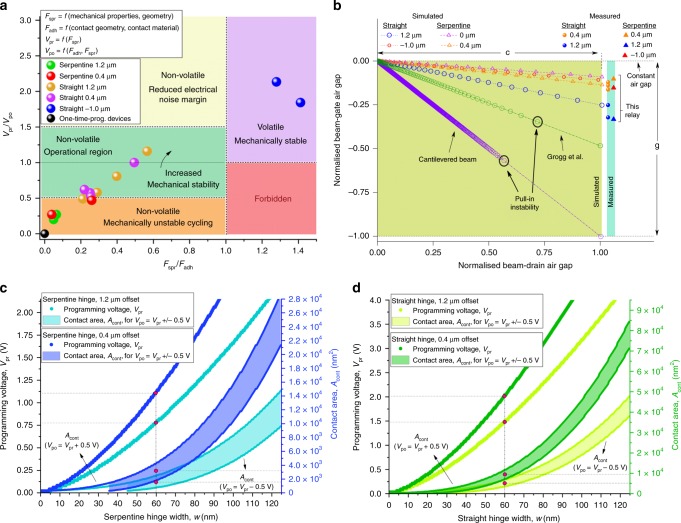
Regimes of operation and scaling study. **a** Operating regimes of NEM relays for serpentine and straight hinges. The condition *F*_adh_ = *F*_spr_ separates the regions where the device is volatile or non-volatile, while *V*_pr_ = *V*_po_ separates the regions where the applied reprogramming voltage causes the device to be overdriven or not. The plot is populated with data points measured in ambient air, room temperature experiments showing NEM relays operating in each regime. **b** Comparison with prior art of uniformity of beam-to-gate airgap *g* vs. beam-to-drain airgap *c* as the relay moves, based on finite-element simulations. The beam-to-gate airgap of the moment-driven relay has under 6% variation for small and negative hinge offsets for the entire range of operation. In contrast, the beam-to-gate airgap decreases by nearly 100% at the beam tip in straight cantilever, bridge, see-saw and crab leg relay architectures unless restricted by the contact dimple. The curved beam relay reported by Grogg et al.^11^ experiences a  ~50% reduction in the airgap at contact. The simulation plot also shows the proposed NEM relay does not exhibit pull-in instability. The measured beam-to-gate displacements after actuation were obtained from SEM scans of the device after beam rotation. This experimental data is plotted on the right. **c**, **d** Scaling study showing variation of programming voltage (i.e. voltage required for closure from a neutral state) with hinge width for actuation and contact airgaps of 60 nm, and contact area needed to achieve a reprogramming voltage within 0.5 V of the programming voltage.

The distinct regimes of operation based on the ratio of experimentally measured programming voltage to pull-out voltage *V*_pr_/*V*_po_ and ratio of hinge spring force to adhesion force *F*_spr_*/F*_adh_ are shown in Fig. 3a. Here *V*_po_ is measured for the first reprogramming cycle, *F*_spr_ is calculated from the analytical model and *F*_adh_ is calculated by the balance of moments at the measured pull-out voltage (see Supplementary Note 2 for model). These measurements were obtained at room temperature in ambient air. The adhesion force in these experiments was consistently higher than in the 200 °C experiments conducted in a vacuum chamber, which can be explained by the presence of capillary forces (the closed environment and high temperature in the 200 °C experiments reduces moisture that can give rise to capillary forces). It should also be noted these measurements required both pull-out and closure events to be recorded, and the electrostatic contribution to the adhesion force would have compounded this effect.

The ratio *F*_spr_*/F*_adh_ defines whether a device is volatile or non-volatile and this boundary is indicated by the vertical line at *F*_spr_/*F*_adh_ = 1 in Fig. 3a. The magnitude of the forces determines the voltage required to break the stiction-based contact. The horizontal line *V*_pr_/*V*_po_ = 1 in Fig. 3a defines the boundary where a reprogramming cycle will not result in over-driving the contact. As discussed above, *V*_po_ >> *V*_pr_ can result in a mechanically unstable device where the over-drive appears to increase the adhesion forces, resulting in progressively higher pull-out voltages. At the other end of the spectrum, when *V*_po_ << *V*_pr_, the electrical noise margin is low, where the propensity is high for induced noise, for example through stray capacitive coupling, to cause a switching upset. Thus, a desirable operating regime can be identified depending on the targeted application. For illustration only, these boundaries are chosen as *V*_po_ = 0.5 *V*_pr_ to *V*_po_ = 1.5 *V*_pr_, and depicted in Fig. 3a.

Finally, some applications require robust one-time programmable memory where the data, once programmed, should not be overwritten. To demonstrate the use of the NEM relay to achieve one-time-programmable behaviour, we have used Cr–Au as the contact material. It is well known that thin layers of gold can be cold-welded at low pressures and temperatures^19^. When thin (~30 nm) layers of Cr–Au were used the contacts microwelded, yielding a reliable one-time programmable device. These relays stayed permanently switched after a single programming cycle and maintained a high drain current (always achieving the set current compliance level of 100 nA), and are shown at the origin of Fig. 3a, i.e. the limit where *F*_adh_, *V*_po_ → *∞*.

## Discussion

We have demonstrated for the first time an electrostatically actuated NEM relay that eliminates pull-in instability without restricting the dynamic range of motion and achieved non-volatile cycling at 200 °C. Table 1 compares the performance of previously reported electrostatically actuated non-volatile relays with the presented relay. To the best of our knowledge, only Soon et al.^14^ have reported more than 1 or 2 reprogramming cycles to date; they demonstrated 11 cycles at 50 °C with a programming voltage of 10 V and reprogramming voltages between ~12 and 15 V. We achieved a programming voltage of ~3.5 V, reprogramming voltages between 7 and 9 V (for cycles without an electrostatic force contributing to the adhesion force), and 42 cycles at 200 °C, before the contact degraded and the on-current decreased below the set current compliance of 1 nA. The devices continued to cycle mechanically until the experiments were halted.

**Table 1:** Comparison of non-volatile relays.

**Reference**	^20^	^13^	^14^	^15^	^16^	**Our device**
Architecture	Out-of-plane See-saw type beam with torsional hinges	In-plane straight cantilevered beam with two gates.	In-plane straight cantilevered fin with two gates.	In-plane straight cantilever with two gates.	In-plane straight cantilevered beam with two gates.	In-plane curved beam with quad gate architecture.
Footprint	500 μm × 25 μm	~3 μm × 2 μm	2 μm beam	0.5 μm beam	1.2 μm beam	~5 μm × 10 μm
Actuation airgap (nm)	450	40	80	25	30	120
Pull-in/programming voltage	3.1 V	1.05 V	10 V	17 V	3–4 V	Various depending on hinge type and offset. 1.6 V for serpentine hinge with 1.2 μm offset.
Reprogramme-ming voltage	N/A	1.95 & 2 V	~12–15 V	25 V	3–4 V	*V*_rep_ = *V*_po_ = 7–8.7 V for device with *V*_pr_ = 3.5 V
Non-volatile cycles	0	2 at room temp.	11 at 50 °C	1 at room temp.	1 at room temp.	42 at 200 °C and 20 at room temp.
Contact material	Ni on Cr–Au	Al	Si doped with As	Si doped with As	Al	Ti
Switching time	Not available.	Not available.	Not available.	Not available.	Not available.	Prog.: 511 ns; and 954 ns for straight, serpentine hinges; reprog.: ~1.5 × prog. time (simulated)
Comment	Stiction recovery in first cycle only. Long beam (0.5 mm) and large gate area to achieve 3 V pull-in.	Reliability not mentioned. Low stiffness of Al (Young’s modulus ~1/3rd that of Si) and small airgap contribute to low pull-in.	Pull-in increases with cycling, failure through microwelding. Similar footprint but much higher device layer thickness (3.5 μm) for a much larger gate area than our device (which has a 300 nm device layer thickness).	Reliability, failure mechanism not discussed.	Complicated approach using charge trapping to alter the pull-in voltage. Reliability and failure mechanism not discussed. Cannot be used at high temperatures due to requirement for charge trapping.	Failure mode is contact resistance increasing. Mechanical failure not observed until experiments were halted, or high overdrive is applied, causing beam to collapse.

The reliability of NEM relays can generally be separated into issues related to the electromechanical operation and issues related to the contact. This work has focused on solving electromechanical issues through operating the relay in a different regime than reported in prior art. The semicircular beam combined with the rotational mode of operation results in a near constant airgap. A finite-element simulation comparison of the maximum beam tip displacement with respect to the actuating gate as the beam tip approaches the drain terminal is shown in Fig. 3b. The displacement of a device with a zero-offset serpentine hinge is shown for reference, as well as displacements of the designs for which measurements were carried out, namely NEM relays with hinge offsets of 0.4, 1.2 and  −1.0 μm. The figure also compares the beam tip movement in the proposed NEM relay to that of a cantilevered beam and the curved beam relay proposed by Grogg et al.^11^. In its programmed state, the maximum change in the airgap for our relay is as low as 6% for small or negative hinge offsets. In the design presented by Grogg et al.^11^ the beam-to-gate airgap reduces to ~50% while in straight cantilever, bridge, see-saw and crab leg relay architectures (e.g. refs. ^5–7,10,12,13^), the gap decreases to close to 100% at the tip, unless restricted by the contact dimple. Furthermore, the sudden jumps in the beam-drain airgap in these devices signal the onset of pull-in instability (crab leg and bridge designs may have different pull-in voltages due to different rates of increase of capacitance with deflection). Our relay, by contrast, does not exhibit such a jump, due to the near constant actuation airgap. Finally, the presented relay moves in plane. Such a design allows a simple fabrication process with a single lithography step and release etch, unlike more complex out-of-plane architectures^5,6,12^.

One consequence of the mechanically stable mode of operation with near constant airgap is that the design is robust against the beam collapsing on the actuating gates. Thus, the actuation airgap of the proposed NEM relay has potential to be more safely scaled than in other architectures to achieve low rotation voltages. Another consequence is that the mechanical hysteresis associated with the different airgaps in closed and open states in traditional architectures can be avoided. Thus, the proposed relay has potential to balance the adhesion and relay spring forces to achieve low reprogramming voltages when used as a non-volatile device. The programming voltages for NEM relays with serpentine and straight hinges with offsets of 1.2 and 0.4 μm, and actuation and contact airgaps of 60 nm are shown in Fig. 3c, d as a function of the hinge width. Based on our experiments we have estimated an average per unit area adhesion force of  ~0.004 nN nm^−2^ for Ti contacts (including the contribution of the electrostatic force from a 2 V drain–source bias). Using this value, also plotted in Fig. 3c, d are estimates of the contact area needed to achieve a reprogramming voltage within 0.5 V of the programming voltage for each type of relay. Within a band, the bottom curve corresponds to *V*_po_ = *V*_pr_ − 0.5 (i.e. smaller contact area and lower adhesion force) and the top curve to *V*_po_ = *V*_pr_ + 0.5 (i.e. larger contact area and higher adhesion force). The mid point of the band corresponds to the point where programming and pull-out voltages are approximately equal. The dimensions of the contact tip required to achieve equal programming and pull-out voltages for a hinge width of 60 nm and device layer thickness of 300 nm, for the four different combinations of hinge type and hinge offset, are given in Table 2.

**Table 2:** Estimated contact area to achieve equal reprogramming and programming voltages (*V*_po_ = *V*_pr_) for devices highlighted in Fig. 3c, d, with *g* = *c* = *w* = 60 nm.

Hinge type	Hinge offset (μm)	*V*_pr_ = *V*_po_	Contact area (nm^2^)	Contact tip length for 300 nm device layer (nm)
Serpentine	1.2	750 mV	1600	5
Serpentine	0.4	1.1 V	2600	9
Straight	1.2	1.5 V	5000	17
Straight	0.4	2 V	10,000	33

In our experiments the lowest programming voltage seen was 1.6 V for a relay with a serpentine hinge having an offset of 1.2 μm. We observed 42 reprogramming cycles at 200 °C in a vacuum ambient for a relay with a serpentine hinge having an offset of 0.4 μm and contact width of 50 nm. The programming voltage for this relay was 3.5 V and reprogramming voltages were  ~7 to 9 V for anticlockwise rotations where electrostatic forces did not contribute to the adhesion force. We achieved 20 cycles in open atmosphere, room temperature experiments with a relay having a straight hinge with offset 0.4 μm and contact width of 50 nm. In all cycling experiments the mode of failure was deterioration of the Ti contacts, and although the relays continued to cycle mechanically, switching events could not be detected electrically. By combining this relay with a contact material that withstands mechanical wear better (such as carbon-based contact materials that have yielded the highest cycling results to date^21–23^ or RuO_2_^24^), NEM relay-based reprogrammable non-volatile memory capable of  >10^4^ reprogramming cycles (typical of harsh-environment capable non-volatile memories) with better data retention and zero current leakage should be achievable. Such a robust relay also has potential for realisation of FPGAs with zero standby power for low throughput applications that require high-temperature capability^8^. Our scaling study reveals that the operating voltage can be reduced to under 1 V by optimising the contact area (Table 2). Under ambient conditions at or near room temperature, the comparative energy efficiency of sub-1 V CMOS processors^25–27^ versus such scaled NEM relay implementations will depend on the throughput requirements and standby characteristics of the application. Applications that require short bursts of low-frequency activity with long periods where the processor is in standby mode are likely to benefit from the zero-leakage property of NEM relays. Thus, applications crucial to reduce dependency on fossil fuels, such as all-electric vehicles and energy-efficient more-electric aircraft, as well as emerging paradigms such as zero-standby power intelligent nodes for the IoT can potentially benefit from this work.

## Methods

### Fabrication

We fabricated a test die containing 66 NEM relays with two different types of hinges, serpentine and straight, three different hinge offsets for each type of hinge, 1.2, 0.4 and  −1 μm and different contact lengths, 100 and 50 nm. To fabricate the relays, a 400 nm-thick positive e-beam resist CSAR-62 was spun on top of a silicon-on-insulator (SOI) wafer with a 300 nm-thick Si device layer and a 400 nm-thick layer of buried SiO_2_ (buried oxide or BOX). The relay pattern was transferred to the resist through e-beam lithography using a Raith Voyager system. After development of the resist, the bare silicon layer was etched by reactive ion etching (RIE), with a mixture of CHF_3_ and SF_6_ gases, using the resist as a mask. The RIE was carried out using an Oxford Instruments PlasmaPro 100 Cobra etching system. The resist was then removed and the structures released by etching the BOX layer with a HF vapour etch. Finally, metal (up to 70–80 nm of Ti for reprogrammable devices and up to 30 nm of Cr–Au for one-time programmable devices) was thermally evaporated on top of the devices. The relevant geometrical parameters are given in Supplementary Table 1.

### Characterisation

The high-temperature measurements were conducted in a vacuum chamber with integrated probes using a Lakeshore EMTTP4 probe station. The test chamber was prepared by flushing it with N_2_ after loading the samples. Subsequently the chamber pressure was reduced to 0.03 mbar while the temperature was increased to 200 °C. Electrical measurements for anticlockwise reprogramming cycles were recorded by applying the actuation voltage simultaneously via an Agilent B1500A semiconductor parameter analyser on gate pair 2 (see Fig. 1c, bottom) with the source grounded. A dc bias of 2 V was applied to drain 2 to monitor the closure event. After closure the actuation voltage is either removed or swept back to zero to generate an *I*–*V* curve for the source-to-drain current. The actuation pattern at Fig. 1c, top was applied for clockwise reprogramming cycles where the beam tip lands on drain 1. The bias on drain 2 was maintained, and the pull-out event was recorded.

The room temperature measurements used a different biasing arrangement in order to be able to independently measure the pull-out and closure events in clockwise reprogramming cycles, shown in Supplementary Fig. 2e. A current compliance of 0.5 and 1 nA was used when applying the drain bias for the Ti-coated reprogrammable non-volatile relays for room temperature and high-temperature measurements respectively, while it was set to 100 nA for the Cr–Au-coated one-time-programmable non-volatile devices. In the atmospheric, room temperature experiments the on-resistance for a given device starts at  ~2 GΩ and quickly deteriorated. The contact resistance in the experiments conducted in the vacuum ambient at high temperature was more stable, with a starting resistance of around 600 MΩ and deteriorating more slowly. The gold-coated contacts used for the one-time-programmable device achieved an on-resistance of  ~6 kΩ.

### Design of the NEM relay

The base architecture of the proposed NEM relay comprises a semicircular beam and four gates (Fig. 1a). The functional behaviour, i.e. whether the relay is volatile or non-volatile, as well as performance-related parameters, such as the initial programming voltage, subsequent reprogramming voltages and switching time, are dependent on the mechanical properties of silicon, actuation and contact airgaps, beam dimensions, hinge design and contact design. The material properties and thickness of the beam (equal to the device layer thickness) are determined by the silicon device layer of the SOI wafer. The actuation and contact airgaps and the beam and hinge widths were held constant over all designs. Thus, the hinge geometry and contact electrode geometries are the main design-related differentiators to achieve different functional and performance characteristics. Relays with two hinge types, serpentine (Fig. 1b) and straight (see Supplementary Note 3 and Supplementary Fig. 2) were fabricated and tested. While the two hinge designs are fixed, the offset of the hinge from the geometric centre of the beam semicircle, which has a direct bearing on the rotational stiffness, is a design variable. This approach, where the principal design parameters are hinge type and hinge offset is similar to having choice of transistor type and freedom to vary the gate width and length in integrated circuit design in a given process technology. The contact material can be chosen to realise one-time-programmable (e.g. Cr–Au) or reprogrammable (e.g. Ti) non-volatile nano relays. The dimensions of the relay designs for the straight and serpentine cantilevers are given in Supplementary Table 1, with the terms defined in Supplementary Fig. 1.

Finally, out-of-plane flexing of the device was investigated using analytical calculations and finite-element simulations. Our calculations show that the out-of-plane to in-plane stiffness ratio for a 300 nm device layer thickness and hinge width of 120 nm results in one to two orders of magnitude difference in the energy required for flexural versus torsional movement. Using Ansys simulations we have estimated that the self weight of the device causes a deflection at the top of the device in the *z* direction of  ~514 fm for a relay with serpentine hinge, and  ~27 fm for a relay with straight hinge. Thus, flexing due to self weight appears negligible. Also, we observed very little out-of plane movement in device suspension or operation.

## Supplementary information

Supplementary Information

## Data Availability

The source data underlying Figs. 1d, e, 3a, b, c and d, and Supplementary Figs. 2d, f, g and 3 are provided as a Source Data file.

## Source data

Source Data
