# Supplementary material for: Nanoelectromechanical relay without pull-in instability for high-temperature non-volatile memory
Source: Nat Commun. 2020 Mar 4;11:1181. doi: 10.1038/s41467-020-14872-2 (PMC7055292; doi:10.1038/s41467-020-14872-2)
Supplement: Supplementary file 1 — Supplementary Information [file 41467_2020_14872_MOESM1_ESM.pdf]

Supplementary Information

Nanoelectromechanical relay without pull-in instability for high-temperature non-volatile memory

Sunil Rana et al.

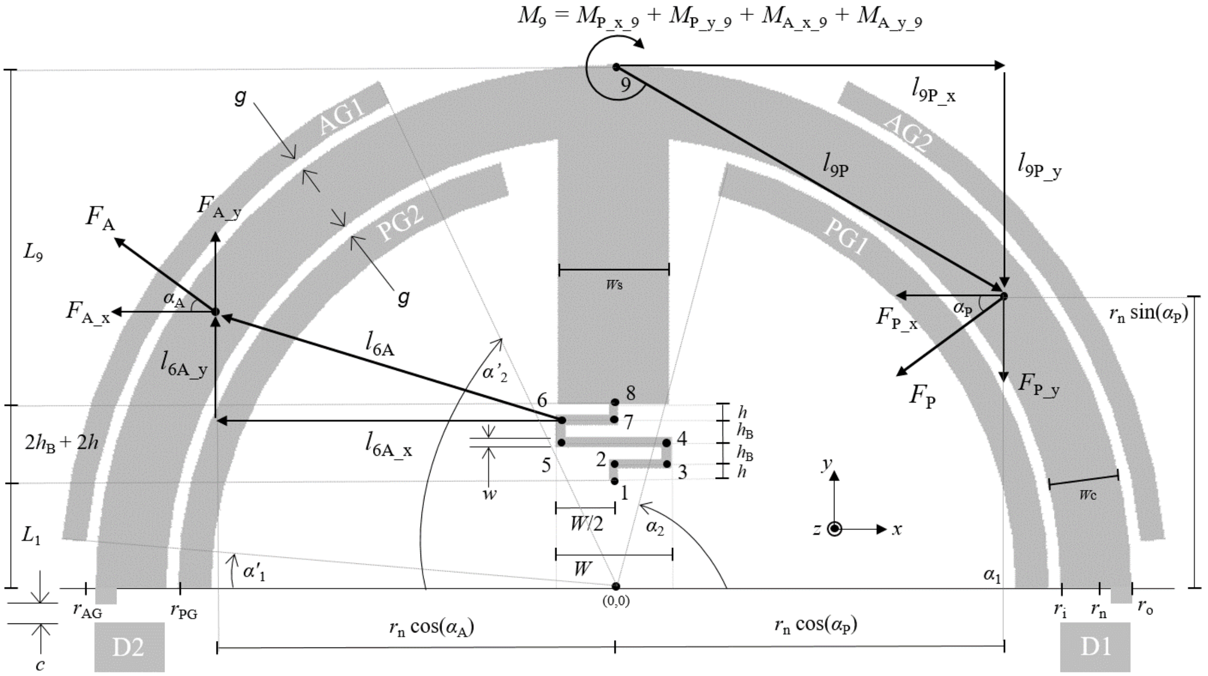

Supplementary Figure 1: **Forces and rotation moments in relay with serpentine hinge.** The serpentine hinge is the general case of a straight hinge. A voltage V is applied simultaneously on the principal and auxiliary gate pair 1. The radially distributed forces are represented by resultant forces F_{P} and F_{A} that act at radius r_{n} that defines the mid-point of the circular arms of the beam and angles α_{P} and α_{A}. These angles are in turn defined by the span angles of the principal and auxiliary gates $\alpha_2 - \alpha_1$ and $\alpha_2' - \alpha_1'$. In this design $\alpha_1 = 0$. The forces are resolved into horizontal and vertical components as shown. The internal bending moments at the top of the beam are also shown. The parameters for the straight hinge are identical, except that points 1 through 8 collapse to one point. Other than the hinge offsets, the geometry of all devices are identical and shown in Table 1.

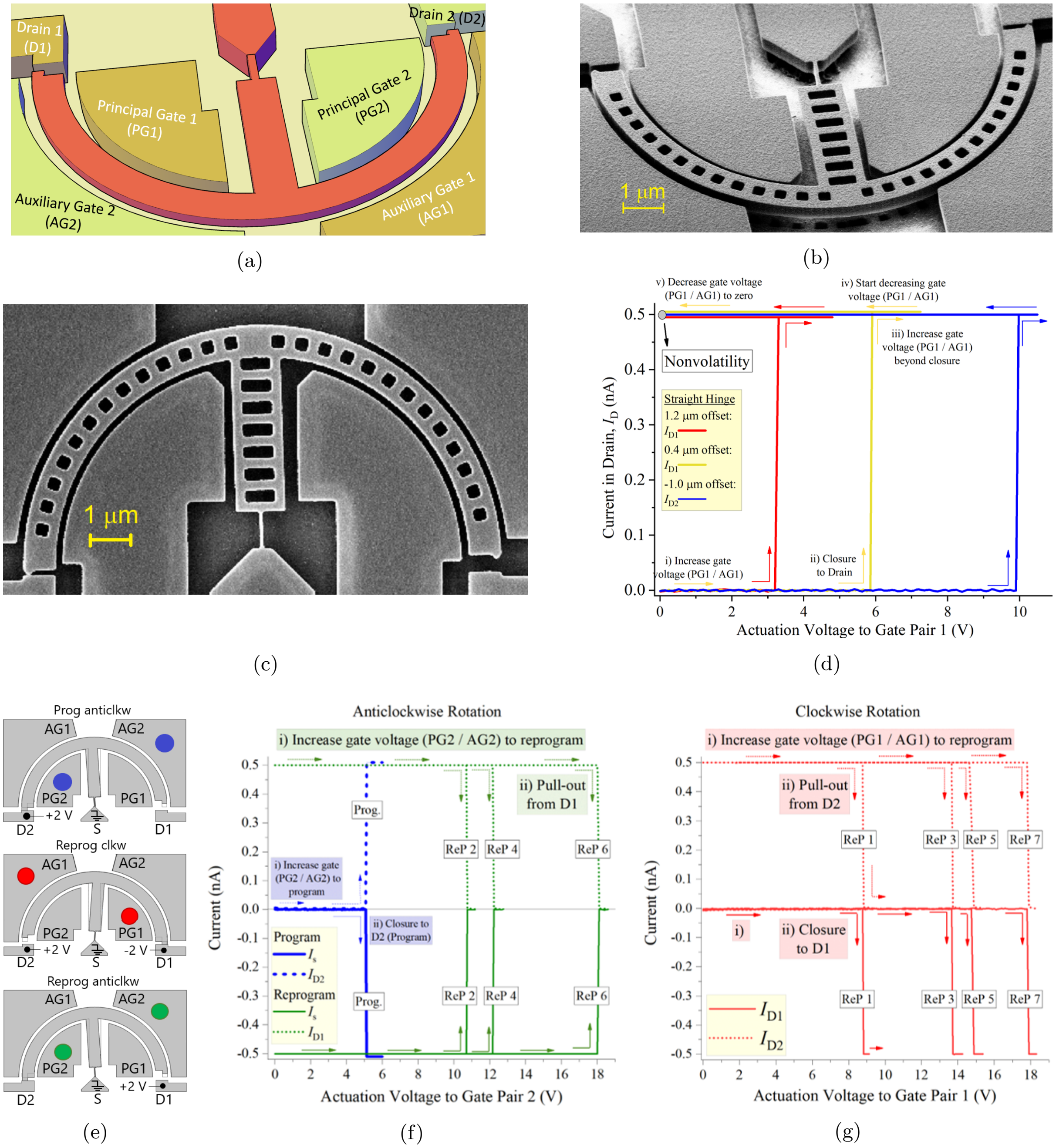

Supplementary Figure 2: **Moment-driven relay with in-plane architecture and straight hinge.** **(a)** Sketch and **(b)** SEM of device with straight hinge; **(c)** bistable nanorelay with 0.4 μm offset (L_1 as defined in Fig. 1), 120 nm gate-to-beam airgap, 100 nm rest-state contact gap and 80 nm hinge width. The gate-to-beam airgap can be seen to have remained nearly constant after rotation. **(d)** Measurements of programming voltage for different hinge offsets (1.2, 0.4, −1 μm) using the actuation pattern of Fig. 2e, top. Switch closure is indicated by the sudden increase in drain current. The beam tip remains in contact with the drain after the actuation voltages are decreased to zero, demonstrating non-volatility. **(e)** Actuation patterns to switch relay, where the circles indicate electrodes where source measurement units are connected; top is used to program the relays anticlockwise from the neutral state; middle is used to reprogram the relays to rotate clockwise, starting from D2; bottom is used to reprogram the relays to rotate anticlockwise, starting from D1. **(f)** First programming cycle and subsequent reprogramming cycles to D2 for the relay shown in 2c. When programmed, current flows between D2 (I_{D2}) and S (I_S). When reprogrammed, the pull-out event is monitored, when I_{D1} and I_S go to zero. **(g)** Clockwise reprogramming cycles (to D1). Both pull-out and closure events are monitored and the current stops flowing through D2 at the same time it starts flowing through D1. Seven consecutive reprogramming cycles are shown in total in Figs. 2f and 2g.

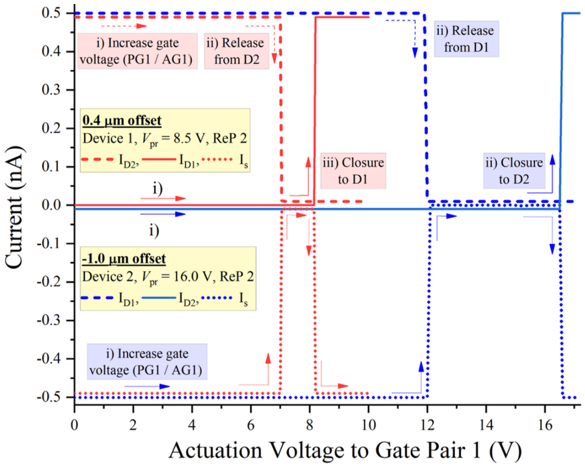

Supplementary Figure 3: **Reprogramming cycles where $V_{\mathbf{po}} < V_{\mathbf{pr}}$** These measurements are for straight hinge devices with different hinge offsets. Cycles are shown for two devices with hinge offsets 0.4 and -1 µm where the voltage required to pull-out of contact is less than the voltage required to rotate the circular beam to make contact with the opposite drain.

Supplementary Table 1: Geometrical parameters for the relay and two types of hinges

All devices		
Thickness (SOI wafer device layer thickness)	t	300 nm
Actuation airgap	g	120 nm
Contact airgap	c	100 nm
Initial angle of Principal Gate	α_1	0°
Final angle of Principal Gate	α_2	75°
Initial angle of Auxiliary Gate	α'_1	5°
Final angle of Principal Gate	α'_2	65°
Inner radius	r_i	5.0 µm
Outer radius	r_o	5.8 µm
Radius of Principal Gate	r_PG	5.92 µm
Radius of Auxiliary Gate	r_AG	4.88 µm
Width of straight central beam	W_s	1.25 µm
Width of semicircular arms	W_c	0.8 µm
Hinge offset	L_1	1.2, 0.4, −1 µm
Straight hinge (St) device only		
Height of straight hinge	L_2	0.84 µm
Width of straight hinge arm	w	75±10 nm
Serpentine hinge (Ser) device only		
Width of serpentine hinge arms	w	75±10 nm
Height of section 1 of serpentine hinge	h	180 nm
Height of section 2 of serpentine hinge	h_B	240 nm
Span of serpentine hinge	W	1.3 µm

Supplementary Note 1: Analytical model for moment-driven operation

The analytical model for our relay has been constructed from first principles as described below. The geometric parameters for a relay with a serpentine design are shown in Supplementary Figure 1. The serpentine hinge is the more general case as the straight hinge is a degenerate version of it where the points 2 through 8 collapse into a single point. When a voltage is applied to principal and auxiliary gate pair 1 (right principal and left auxiliary gates), electrostatic forces are distributed radially across the respective airgaps and can be represented by resultant lumped forces as shown. The magnitude of the force vectors for an applied voltage V can be determined from the capacitance of the two cylindrical capacitors defined by the circular arms and the actuating electrodes:

$$F = \frac{\alpha \epsilon_0 t V^2}{2b \left[\ln(b/a)\right]^2} \tag{1}$$

where ϵ_0 is the free space permittivity and t is the thickness of the relay beam and gates. For the inner capacitance, b is the inner radius of the circular beam r_i, a the radius of the principal gates r_PG and $\alpha = \alpha_2 - \alpha_1$. For the outer capacitance, $b = r_\mathrm{AG}$, $a = r_\mathrm{o}$ and $\alpha = \alpha_2' - \alpha_1'$.

The force vectors F_P and F_A (see Supplementary Figure 1) can be resolved into x and y components. The x force components $F_\mathrm{P\_x}$ and $F_\mathrm{A\_x}$ act in synchrony to move the beam to the left while the y force components, $F_\mathrm{P\_y}$ and $F_\mathrm{A\_y}$ act against each other. The direct effect of the x force vector components $F_\mathrm{P\_x}$ and $F_\mathrm{A\_x}$ upon the top of the beam is to displace it in the horizontal direction. Each of the four force vector components also imparts bending moments on the hinge and beam that are clockwise or anticlockwise based on the location of the hinge anchor point. The moments at points 1 through 9 (see Supplementary Figure 1) are defined in equations (2) through (5):

$$M_{\mathrm{P\_x}(i)} = -l_{i\mathrm{P\_y}} F_\mathrm{P\_x} \text{ where } F_\mathrm{P\_x} = -F_\mathrm{P} \cos \alpha_\mathrm{P} \tag{2}$$

$$M_{\mathrm{P\_y}(i)} = l_{i\mathrm{P\_x}} F_\mathrm{P\_y} \text{ where } F_\mathrm{P\_y} = -F_\mathrm{P} \sin \alpha_\mathrm{P} \tag{3}$$

$$M_{\mathrm{A\_x}(i)} = -l_{i\mathrm{A\_y}} F_\mathrm{A\_x} \text{ where } F_\mathrm{A\_x} = -F_\mathrm{A} \cos \alpha_\mathrm{A} \tag{4}$$

$$M_{\mathrm{A\_y}(i)} = l_{i\mathrm{A\_x}} F_\mathrm{A\_y} \text{ where } F_\mathrm{A\_y} = F_\mathrm{A} \sin \alpha_\mathrm{A} \tag{5}$$

with $\alpha_\mathrm{P} = \frac{\alpha_1+\alpha_2}{2} + \alpha_1$ and $\alpha_\mathrm{A} = \frac{\alpha_1'+\alpha_2'}{2} + \alpha_1'$ and where $l_{i\mathrm{P}} = l_{i\mathrm{P\_x}} + l_{i\mathrm{P\_y}}$ and $l_{i\mathrm{A}} = l_{i\mathrm{A\_x}} + l_{i\mathrm{A\_y}}$ are the sum of x and y vector components of the direction vector (or the lever arm vector), defined as the vector connecting the point i of the device to the point where the electrostatic force is applied. Our adopted sign convention is that a negative moment causes a clockwise rotation of the device, and an anticlockwise moment is treated as positive.

The displacement of the tip of the beam as it rotates can be calculated by consideration of the strain energy. The internal strain energy of a spring U_{ji} is calculated as the sum of individual strain energies resulting from axial loading, U_AL, transverse shear loading, U_TS, and bending moments, U_BM, acting in a beam segment $j - i$ anchored at j, as described in Supplementary Figure 1. The total internal strain energy of the spring is therefore calculated as the sum of the individual strain energies:

$$U_{ij} = U_\mathrm{AL} + U_\mathrm{BM} + U_\mathrm{TS} \tag{6}$$

where

$$U_\mathrm{AL} = \int_0^L \frac{N^2}{2AE} dy \ , \ \ U_\mathrm{TS} = \int_0^L \frac{k_\mathrm{x,y} {V_\mathrm{S}}^2}{2GA} dy \text{ and } \quad U_\mathrm{BM} = \int_0^L \frac{M^2}{2EI} dy \tag{7}$$

in a beam segment with length L, cross-sectional area A, modulus of elasticity E, shear modulus G, second moment of area I and spring constant $k_\mathrm{x,y}$ in direction x when the force is applied in the y direction for a rectangular cross-section having axial load N, shear force V_S and internal bending moment M. In (7) the axial and shear loads N and V_S are the x or y components of the electrostatic forces depending on whether the considered beam segment is vertical or horizontal. The bending moment M cited in equation (7) is the sum of the moments created by these same forces and described in equations (2)

through (5). The moments of area, I, for the different segments are calculated for the precise geometry used for fabrication, including release etch holes in the central and circular arms. Given the strain energy in equation (6) the displacements of the tip i of each individual beam in the x and y directions, δ_x and δ_y, are calculated by Castigliano's second theorem, giving:

$$\delta_{\mathrm{x},i} = \frac{\partial U_{ji}}{\partial F_{\mathrm{x}}} \ , \ \ \delta_{\mathrm{y},i} = \frac{\partial U_{ji}}{\partial F_{\mathrm{y}}}. \tag{8}$$

This is applicable for any segment including not only the sections in the serpentine hinge but also the vertical section (segment 8-9). Equation (8) can be solved using the relations in equations (1) through (7) to yield a closed-form solution for the displacement of each point i. The angle of rotation associated with the displacement of point i is given by

$$\theta_{i,\mathrm{v}} = tan^{-1}\left(\frac{\delta_{\mathrm{x}\_i}}{L_i - \delta_{\mathrm{y}\_i}}\right) \ , \ \ \theta_{i,\mathrm{h}} = tan^{-1}\left(\frac{\delta_{\mathrm{y}\_i}}{L_i - \delta_{\mathrm{x}\_i}}\right) \tag{9}$$

for vertical (points $i = 2, 4, 6, 8$ and 9) and horizontal (points $i = 3, 5$ and 7) beams, respectively (note that the straight hinge devices only have vertical points $i = 2, 3$). The total angle of rotation of the device is calculated as the sum of the angles of rotation of each individual segment ($\theta = \sum_i \theta_i$). The angle determines the length of the arc described by the tip of the bifurcated beam of the device as a result of the hinge deformation, and is given in eq. (10):

$$s = \sqrt{(L_1^2 + r_{\mathrm{o}}^2)}\ \theta. \tag{10}$$

This first principle model has been checked against Ansys simulations for different relay designs comprising the two types of hinges and the different hinge offsets and dimensions used for the experiments, as detailed in Table 1. The model slightly overestimates V_{pr} for a given geometry compared to Ansys (due to linear strain and lumped force approximations). To increase the accuracy in Fig. 2b, we introduced a constant empirical calibration factor that reduces the hinge width by 20 nm.

Supplementary Note 2: Analytical model for pull-out event

The pull-out event is modelled by considering the torque balance at the top of the beam ($i = 9$ for the serpentine hinge shown in Fig. 1). For a non-volatile relay, pull-out occurs when the combined moments from the spring force M_{spr} and electrostatic force M_{e} caused by actuating the opposing pair of gates overcome the moment caused by the adhesion force. Assuming a starting position where the device is rotated to drain 1, this torque balance for point 9 is given by

$$-M_{9\_\mathrm{spr}} - M_{9\_\mathrm{e}} + M_{9\_\mathrm{adh}} \geqslant 0 \tag{11}$$

where M_{9_spr} is calculated by the sum of the moments in equations (2) through (5) for the voltage required for closure (using the programming voltage) to drain 1, where M_{9_e} is calculated by the sum of the moments in equations (2) through (5) for the voltage required for release and $M_{9\_\mathrm{adh}} = r_{\mathrm{o}}F_{\mathrm{adh}}$.

Supplementary Note 3: NEM relay with straight hinge design

In this work NEM relays with straight hinges have also been fabricated and characterised. All of the measurements reported in this section were carried out at room temperature, under atmospheric conditions. The schematic and SEM image of a NEM relay with a straight hinge are shown in Supplementary Figure 2a and Supplementary Figure 2b. Near constant inner and outer airgap can be seen after (anticlockwise) rotation of the beam in Supplementary Figure 2c. To investigate the influence of the hinge offset on the required programming voltage in devices with straight hinges, relay designs with three different offsets were fabricated. Programming measurements for these NEM relays were carried out using the actuation pattern of Supplementary Figure 2e, top. These waveforms are shown in Supplementary Figure 2d where the following regimes can be identified in each waveform: i) The actuation voltage to gate pair 1 (PG1 / AG1) is increased; ii) when the actuation voltage on gate pair 1 reaches the closure voltage, the beam tip makes contact with D1 (positive hinge offset), or D2 (negative hinge offset) shown by the step increase in the current flowing through the drain and the beam; iii) the actuation voltage on gate pair 1 is increased

by around 2 V beyond the closure voltage; iv) the actuation voltage on gate pair 1 is then decreased, with the drain current continuing to flow all the while; v) the actuation voltage on gate pair 1 reaches zero, and the drain current continues to flow at the set current compliance, demonstrating non-volatility. These regimes are illustrated by the arrows in Supplementary Figure 2d associated with each waveform. It can be seen that the programming voltage decreases with increasing hinge offset, same as in nanorelays with a serpentine hinge.

In order to demonstrate reprogrammable non-volatile functionality, the actuation sequences shown in Supplementary Figure 2e were applied. Starting from the neutral position (as fabricated) the device was first rotated anticlockwise to D2 by applying actuation voltages to the gate pair shown at the top (blue pattern) with the source grounded. A dc bias of 2 V was applied to D2 in order to monitor closure. The following regimes are illustrated for the programming (blue) waveform in Supplementary Figure 2f: i) The actuation voltage to gate pair 2 (PG2 / AG2) is increased; ii) when the actuation voltage on gate pair 2 reaches the programming voltage, the beam tip makes contact with drain 2 (D2), shown by the step increase in the current I_{D2} flowing through D2 and S, where $I_{\mathrm{D2}} = -I_{\mathrm{S}}$.

Once programmed, the actuation voltage was removed from gate pair 2 and applied to gate pair 1 to reprogram through clockwise rotation as per the actuation pattern shown in Supplementary Figure 2e, middle (i.e. red pattern). The clockwise reprogramming cycles are shown in Supplementary Figure 2g, where the device starts off being in contact with D2. The following regimes are shown for each waveform: i) The actuation voltage to gate pair 1 (PG1 / AG1) is increased; ii) when the actuation voltage on gate pair 1 reaches the pull-out voltage, a step decrease in I_{D2} can be seen. For these relays pull-out is immediately followed by closure to the opposite drain, which can be seen by the step increase in I_{D1}.

After a clockwise reprogramming cycle, the actuation pattern shown in Supplementary Figure 2e, bottom (i.e. green pattern) was used to achieve anticlockwise rotation. These waveforms are shown in Supplementary Figure 2f in green, where the device starts off being on contact with D1. The following regimes are illustrated: i) The actuation voltage to gate pair 2 (PG2 / AG2) is increased; ii) when the actuation voltage on gate pair 2 reaches the pull-out voltage, a step decrease in I_{D1} and I_{S} ($I_{\mathrm{D1}} = -I_{\mathrm{S}}$) can be seen. Closure to D2 is not monitored, but subsequently confirmed by an I-V curve. Seven consecutive reprogramming cycles are plotted in total in Supplementary Figure 2f and Supplementary Figure 2g, with the initial cycle named the 'Prog' cycle and subsequent cycles named 'ReP 1', 'ReP 2' and so on. For this device the initial rotation voltage was 5.1 V ('Prog') with the first pull-out ('ReP 1') occurring at 8.7 V.

In these actuation patterns a drain bias was always present on pull-out, which likely added an electrostatic force component to the adhesion force (our experiments with serpentine hinge devices revealed that the drain bias consistently increases the pull-out voltage by $\sim$ 1–2 V, see Figs. 1d and 1e in main article). Thus, the reprogramming voltages are high with respect to the programming voltage and the device is heavily over-driven at the point of pull-out, increasing the impact force with which the moving electrode lands on the opposite drain. This appears to increase the effective area of the nano contact, likely increasing van der Waal surface forces [1] and causing the reprogramming voltage to further increase. This effect appears to be perpetuated for a few cycles until it levels off to a certain extent (ReP 7), which we speculate is because the actuation voltage is high enough that any further increase in the adhesion force is relatively small compared to the actuating electrostatic force at that point.

Supplementary Note 4: Reprogramming cycles where the pull-out voltage is less than the closure voltage

The regime of operation where $V_{\mathrm{po}} < V_{\mathrm{pr}}$ is important for preventing gate overdrive that can result in unpredictable reprogramming voltages. It is also critical in characterising the adhesion force for a given contact design and area without having to include the effect of the impact force as a function of gate overdrive. Reprogramming cycles for this regime are shown in Supplementary Figure 3, for two NEM relays with straight hinges and hinge offsets of 0.4 and -1 µm. These are non-volatile devices initially in contact with either drain 2 (device with a positive hinge offset) or drain 1 (device with a negative hinge offset). The following regimes are illustrated in the waveform (red) for the relay with a positive hinge offset: i) The actuation voltage to gate pair 1 (PG1 / AG1) is increased, as a current flows through D2 and S ($I_{\mathrm{D2}} = -I_{\mathrm{S}}$), but not D1 (the beam tip is connected to D2); ii) when the actuation voltage on gate pair 1 reaches the pull-out voltage, a step decrease in I_{D2} and I_{S} can be seen; iii) as the actuation

voltage on gate pair 1 is further increased and reaches the closure voltage, a step increase in I_{D1} and I_{S} ($I_{\mathrm{D1}} = -I_{\mathrm{S}}$) occurs.

The following regimes are illustrated in the waveform (blue) for the relay with a negative hinge offset: i) The actuation voltage to gate pair 1 (PG1 / AG1) is increased, as a current flows through D1 and S ($I_{\mathrm{D1}} = -I_{\mathrm{S}}$), but not D2 (the direction of rotation is reversed for relays with negative offsets); ii) when the actuation voltage on gate pair 1 reaches the pull-out voltage, a step decrease in I_{D1} and I_{S} can be seen; iii) as the actuation voltage on gate pair 1 is further increased and reaches the closure voltage, a step increase in I_{D2} and I_{S} ($I_{\mathrm{D2}} = -I_{\mathrm{S}}$) occurs.

The plots show that each device is shown to pull out of contact from the drain at a lower voltage than that required for rotation to make contact with the opposite drain. Device 1 in Supplementary Figure 3 with a 0.4 µm hinge offset had a programming voltage of 8.5 V, and pulled out in the second reprogramming cycle at 7 V. The reprogramming voltage for cycle 2 was 8.2 V. Differences in clockwise and anticlockwise programming voltages could be due to asymmetries in actuation gaps and alignment arising from patterning and etch non-uniformities as well as intrinsic stresses as the device is suspended. Device 2 in Supplementary Figure 3 had a −1 µm hinge offset and a programming voltage of 16 V. The pull-out and reprogramming voltages in the second reprogramming cycle were 12 and 16.3 V. The regime of operation where $V_{\mathrm{po}} < V_{\mathrm{pr}}$ was only observed for the stiffer devices as a higher stiffness contributes to a lower V_{po}.

Supplementary References

[1] J. N. Israelachvili, *Intermolecular and surface forces.* Academic press, 2011.
